# Molecular Characterization of a First-in-Human Clinical Response to Nimesulide in Acute Myeloid Leukemia

**DOI:** 10.3389/fonc.2022.874168

**Published:** 2022-06-08

**Authors:** Victória Tomaz, Karina Griesi-Oliveira, Renato D. Puga, Bruno J. Conti, Fabio P. S. Santos, Nelson Hamerschlak, Paulo V. Campregher

**Affiliations:** ^1^ Experimental Research Laboratory, Hospital Israelita Albert Einstein, São Paulo, Brazil; ^2^ Medicina Personalizada, Grupo Pardini, São Paulo, Brazil; ^3^ Centro de Hematologia e Oncologia Familia Dayan-Daycoval, Hospital Israelita Albert Einstein, São Paulo, Brazil

**Keywords:** acute myeloid leukemia, anti-inflammatory agents, nimesulide, RNA-Seq, autophagy

## Abstract

Acute myeloid leukemia (AML) is a hematologic malignancy associated with high morbidity and mortality. Here we describe a case of a patient with AML who presented a partial response after utilization of the non-steroidal anti-inflammatory drug nimesulide. The response was characterized by complete clearance of peripheral blood blasts and an 82% decrease of bone marrow blasts associated with myeloblast differentiation. We have then shown that nimesulide induces *in vitro* cell death and cell cycle arrest in all AML cell lines (HL-60, THP-1, OCI-AML2, and OCI-AML3). Weighted Correlation Network Analysis (WGCNA) of serial whole-transcriptome data of cell lines treated with nimesulide revealed that the sets of genes upregulated after treatment with nimesulide were enriched for genes associated with autophagy and apoptosis, and on the other hand, the sets of downregulated genes were associated with cell cycle and RNA splicing. Serial transcriptome of bone marrow patient sample confirmed the upregulation of genes associated with autophagy after the response to nimesulide. Lastly, we demonstrated that nimesulide potentiates the cytotoxic *in vitro* effect of several Food and Drug Administration (FDA)-approved chemotherapy drugs used in AML, including cytarabine.

## Introduction

Despite therapeutic advances in recent years, acute myeloid leukemia (AML) is still associated with high morbidity and mortality, with most patients succumbing to the disease (1, 2). AML treatment arsenal comprises chemotherapy, hypomethylating agents, targeted therapies, and hematopoietic stem cell transplantation (HSCT) (3). While remission can be induced in most patients, the only curative approach for the majority of individuals with AML remains HSCT. Nevertheless, the morbidity associated with HSCT and the median age at diagnosis of 68 years make a significant proportion of patients ineligible for such treatment (4, 5). Therefore, new treatment strategies are needed in AML.

Drug repurposing, that is, the identification of new uses for existing drugs, is a strategy with the potential to improve treatment outcomes with significantly cheaper, faster, and safer preclinical and clinical development protocols (6). Several investigators have already explored such strategy with successful examples, such as the repositioning of thalidomide for the treatment of multiple myeloma and the subsequent development of the thalidomide analog lenalidomide, the use of rituximab for rheumatoid arthritis treatment, and the use of aspirin, a COX inhibitor, as secondary prevention in colorectal cancer (7–10).

There is preclinical evidence that non-steroidal anti-inflammatory drugs (NSAIDs) have anti-proliferative and pro-apoptotic effects in AML cell lines and xenograft models (11–13). In addition, studies have shown that the COX-2 inhibitor nimesulide, in addition to inducing *in vitro* leukemic cell death, also induces myeloid differentiation (12). There is also evidence that glucocorticoids associated with chemotherapy may have a role in the treatment of specific subsets of AML, with at least one study demonstrating improved overall and disease-free survival in patients with hyperleukocytic AML (14).

In this article, we report the first-in-human clinical response to nimesulide in AML and provide insights into the mechanisms of action involved in this effect through whole exome, transcriptome, and cell culture experiments.

## Materials and Methods

### Patient Biological Sample

Peripheral blood and bone marrow samples were obtained from an AML patient after the informed consent form was signed. Blood count was performed on an XP-300 hematology analyzer (Sysmex, Kobe, Japan), and the bone marrow smear was processed according to the standards of the diagnostic medical laboratory. Bone marrow mononuclear cells (BMMC) were obtained using Ficoll-Paque (Sigma-Aldrich, St. Louis, MO, USA) and used for immunophenotyping, DNA, and total RNA extraction for whole-exome and transcriptome sequencing (**Supplementary Methods**).

### Cell Culture

The leukemic cell lines HL-60, THP-1, OCI-AML2, and OCI-AML3 were obtained from American Type Culture Collection (ATCC; Manassas, VA, USA) or Leibniz Institute DSMZ (German Collection of Microorganisms and Cell Cultures GmbH) and cultured in RPMI-1640 medium containing 10% fetal bovine serum, 2 mM of l-glutamine, 100 U/ml of penicillin, and 100 μg/ml of streptomycin (all Gibco, Grand Island, NY, USA) at 37°C in a 5% CO_2_-humidified atmosphere.

### Compounds

The drugs nimesulide (Oficinal Pharmacy, São Paulo, SP, BR), prednisolone (Biossynthetic, São Paulo, SP, BR), and cytarabine (Libbs, , São Paulo, SP, BR) were used in the cellular experiments. Nimesulide was diluted in dimethyl sulfoxide (DMSO) in a 10-mM stock solution, and prednisolone and cytarabine, presented in liquid form, were diluted in RPMI medium immediately before being applied to the cells. Food and Drug Administration (FDA)-approved drug library (Selleck Chemicals, Houston, TX, USA) was used for drug screening in combination with nimesulide.

### Flow Cytometry Assays

The eBioscience™ Annexin V Apoptosis Detection Kit FITC (Invitrogen, Carlsbad, CA, USA) was used for the detection of early and late apoptosis in treated cell lines, following the manufacturer’s instructions. For the cell cycle analysis, treated cells were washed with cold phosphate-buffered saline (PBS) twice and fixed with 70% cold ethanol at 4°C overnight. After cells were washed again with PBS twice, they were incubated with PBS containing 10 mg/ml of RNase at 37°C for 30 min, followed by DNA staining with 1 mg/ml of propidium iodide (all Invitrogen). After being left in a dark place at 4°C for 10 min, the cell DNA content was evaluated using a flow cytometer to analyze the distribution of DNA content in cell cycle phases. All experiments were performed using flow cytometer FACScan Fortessa (BD Biosciences, San Jose, CA, USA) and recorded at least 10,000 events/sample. The analysis of the experiments was performed by FlowJo software.

### Drug Screening and Cell Viability Assay

To identify potential effects of small molecules in combination with nimesulide, an FDA-approved drug library was purchased from Selleck Chemicals for a high-throughput drug screening with selected drugs to assess the roles of drugs in cells lines. The following drugs approved for the treatment of AML were selected: azacitidine (1 μM), gilteritinib fumarate (0.1 μM), cladribine (10 μM), doxorubicin hydrochloride (6.7 μM), mitoxantrone hydrochloride (0.33 μM), fludarabine (0.09 μM), thioguanine (1.78 μM), and decitabine (0.5 μM). All drugs were diluted in RPMI 1640 culture medium (Gibco) to obtain all the above concentrations, based on the lowest concentration found in the literature. Cell viability was analyzed using the CellTiter-Glo^®^ Luminescent Cell Viability Assay (Promega, Madison, WI, USA) according to the manufacturer’s instructions. After 24 h of incubation of cell line with drugs alone or in combination with nimesulide, the cells were incubated for 10 min with Cell Titer-Glo reagent and then assayed by a luminescence plate reader. The ATP content of cells was calculated using the ATP standard curve in a preconfigured CellTiter-Glo protocol on GloMax^®^ Discover Microplate Reader (Promega).

### RNA Extraction and Sequencing

RNA was extracted with RNeasy Mini Kit (Qiagen, Hilden, Germany), and the library preparation and sequencing were performed at the Oklahoma Medical Research Foundation Genomics Core (USA) (**Supplementary Methods**). Sequencing was performed for groups treated with nimesulide and prednisolone alone, in combination, and for the control group (DMSO), in two independent experiments. Three samples from the patient were also sequenced at different times of the disease: at diagnosis (transcriptome 1), on D+21 after the start of nimesulide (transcriptome 2), and on D+63 after the start of nimesulide (transcriptome 3). The bioinformatics pipeline used for processing the sequenced samples is available in the **Supplementary Material**.

### Gene Co-Expression Network Analysis

The Weighted Correlation Network Analysis (WGCNA) package from R (15) was employed to construct the gene co-expression network and identify the co-expression modules related to the treatments. For the construction of the networks and module assignment, the parameters adopted, using the function blockwiseModule, for the two analyzes were as follows: power = 20, networkType = “signed”, minModuleSize = 150, mergecutHeight = 0.15 verbose = 6, minKMEtoStay = 0.5, nThreads = 24, and maxBlockSize = 20,000. The top ~10,000 genes with the highest variance among those genes considered as expressed were selected. For each module, the gene expression levels of each sample were summarized into an eigengene value, which was then used to assess the correlation of a module with proposed treatments in the leukemic cell lines. The significance value for these correlations was considered the value of p < 0.05 divided by the number of modules generated for each experiment. For experiment 1, p-value ≤0.0022 was considered, and for experiment 2, p-value ≤0.0027. The function userListEnrichment from WGCNA was used to evaluate the overlap between the modules identified in experiments 1 and 2. The functional enrichment analysis of the differentially expressed modules of the WGCNA was performed using the Database for Integrated Annotation, Visualization and Discovery (DAVID) v6.8, and for the protein–protein enrichment test of the WGCNA modules, the analysis of functional enrichment of protein–protein interaction networks (STRING) v11.0 was used. The functional categories observed were from the Gene Ontology (GO) Consortium, and they were related to biological processes, cellular components, and molecular functions. The KeggCharts, which show the distribution of genes between the Kyoto Encyclopedia of Genes and Genomes (KEGG) biochemical pathways, were also analyzed.

### Analysis of the Patient’s Transcriptomes

The strategy used to compare the molecular findings from cell line transcriptomes with the patient’s transcriptomes was the CAMERA (16) package. The transcriptome data at time points 2 and 3 of the patient were compared with the transcriptome data at time point 1 in order to generate an expression difference ratio between these times. These expression ratio values were used to rank the genes by foldchange, from the highest to lowest, and then the CAMERA package was used, which performs a differential expression analysis, in which a p-value is assigned, in order to identify whether genes from a given set are assigned non-randomly within this ranking. This analysis was performed comparing the two patient rankings with the modules differentially expressed in the WGCNA.

### Statistical Analysis

For flow cytometry assays, ANOVA statistical test was used, followed by Tukey’s test for multiple comparisons. The statistical computations were conducted using GraphPad Prism 7.0, and the significance level adopted was 5% (p < 0.05). For the bioinformatics analysis, statistical methods specific to each computer program were used.

## Results

### Clonal Dynamics and Partial Response of a Patient With Acute Myeloid Leukemia After Use of Nimesulide

**Figure 1:**
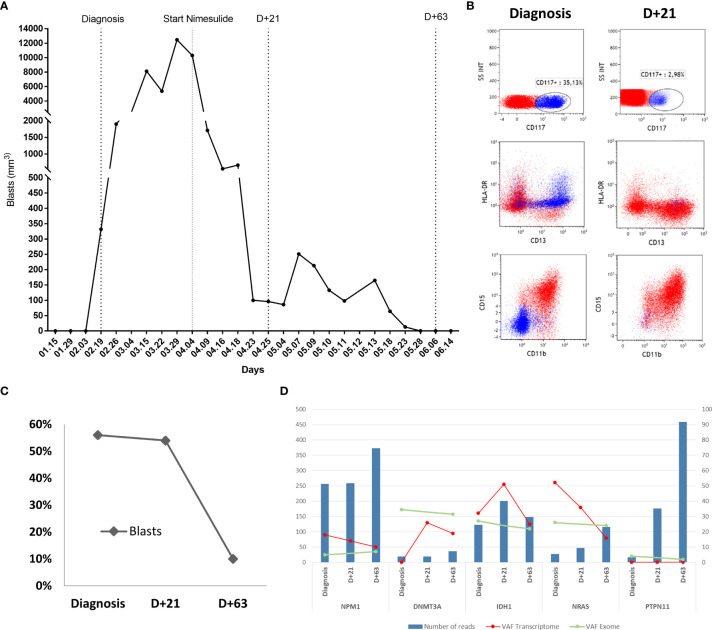
Patient's response to the use of nimesulide. **(A)** Graph of absolute values of blasts in the patient’s peripheral blood at diagnosis and after starting nimesulide use. **(B)** Dot plot graphs of the expression of HLA-DR, CD117, CD15, CD13, and CD11b markers in the patient's bone marrow leukemic blast population at diagnosis and D+21 after starting nimesulide. **(C)** Percentage of blasts in the patient's bone marrow at diagnosis and after days of nimesulide use (D+21 and D+63). **(D)** Graph showing the total number of reads (reads coverage) of NPM1, DNMT3A, IDH1, NRAS, and PTPN11 in the patient's three transcriptomes (Diagnosis, D+21, and D+63) represented in the bars in blue and the allelic frequency of variants (VAF) of the respective mutations from transcriptomes (red line) and whole-exome sequencing (green line). The left y-axis represents the values of the numbers of reads (blue bars), and the right y-axis refers to the transcriptome (red line) and exome (green line) VAF values.

A 52-year-old female was evaluated for asthenia, and her complete blood counts revealed a hemoglobin level of 10.1 g/dL, white blood cell (WBC) count of 4,740/mm^3^ (7% blasts, 1% metamyelocytes, 4% bands, 52% neutrophils, 30% lymphocytes, and 6% monocytes) and a platelet count of 139,000/mm^3^. Bone marrow evaluation was compatible with the diagnosis of AML with 56% blasts on bone marrow smear. Blast immunophenotyping was positive for CD117, CD64, CD36, CD33, HLA-DR, and negative for CD34. Cytogenetics analysis revealed a diploid karyotype (46, XX), and whole-exome sequencing revealed the following driver mutations NPM1:NM_002520:c.861_862insTGCT:p.L287fs, DNMT3A:NM_175629:c.1658delA:p.N553fs, IDH1:NM_005896:c.G395A:p.R132H, NRAS : NM_002524:c.G38T:p.G13V, and PTPN11:NM_080601:c.G794A:p.R265Q with wild-type FLT3 and CEBPA. Her final diagnosis was AML with mutated NPM1, and the European LeukemiaNet risk stratification was a favorable risk (17). The patient decided to receive only palliative care without specific AML treatment. Eleven days after her initial diagnosis, the patient developed gingival infiltration that required a minor oral procedure, and nimesulide 200 mg/day was prescribed for pain control. A few days after the start of nimesulide, she presented a progressive decrease in her peripheral blood blast count and improvements in her stamina. She had also been using prednisolone 30 mg/day for 3 months due to idiopathic knee arthritis. Therefore, the medical team decided to keep both drugs in a continuous fashion given the unexpected clinical response. Seventy-two days after the start of nimesulide treatment, her peripheral blood blast count went from 12,000 blasts/mm^3^ to undetectable levels (**Figure 1A**). A bone marrow evaluation 21 days after nimesulide start revealed two immunophenotypic distinct blast populations, with 27% blasts presenting the same phenotype found at diagnosis and 27% of a second blast population characterized by increased expression of CD13, CD15, and CD11b and negativity for HLA-DR and CD117 (**Figure 1B**), compatible with blast differentiation. A third bone marrow evaluation 63 days after nimesulide revealed only 10% blasts with the same phenotype seen at diagnosis, characterizing a partial response (**Figure 1C**). In order to evaluate the clonal dynamics of mutations associated with the response to nimesulide, we performed whole-transcriptome sequencing at diagnosis and at two different times after the start of nimesulide (D+21 and D+63) and whole-exome sequencing at diagnosis and after the start of nimesulide (D+63).

The analysis of bone marrow whole exome at diagnosis suggests an ancestral clone harboring the DNMT3A mutation with an allele frequency of 34.5%; a second subclone with IDH1 and NRAS with allele frequencies of 27% and 26%, respectively; and the third subclone with NPM1 and PTPN11 with allele frequencies approximately 5% (**Figure 1D**). When comparing the sequencing at diagnosis and on D+63, we observe the maintenance of all five mutations with similar allele frequencies, despite an 82% reduction in the blast percentage (**Figure 1D**), strongly suggesting myeloid differentiation, in agreement with the immunophenotyping data. Transcriptome analysis of bone marrow at diagnosis and on D+21 and D+63 reveals a heterogeneous pattern of expression change of the mutated transcripts, with a progressive increase in the expression of mutated DNMT3A, a progressive decreased expression of mutated NPM1 and NRAS, and an initial increase followed by decreasing expression of mutated IDH1 (**Figure 1D**). Expression of mutated PTPN11 was not detected at any time point.

After 3 months of nimesulide treatment, peripheral blood blasts started increasing again, and the patient passed away 8 months after diagnosis due to progressive AML.

### Anti-Inflammatory Compounds Induce Cell Death and Cell Cycle Arrest in Acute Myeloid Leukemia Cell Lines

**Figure 2:**
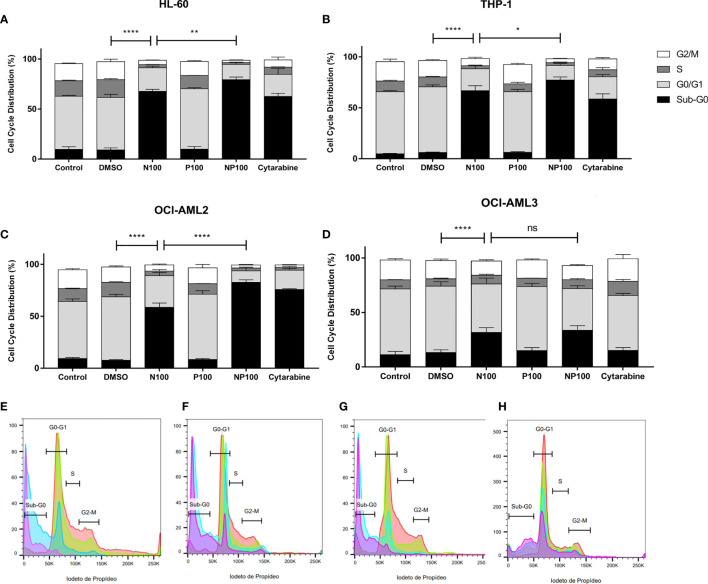
Effect of nimesulide, prednisolone, and cytarabine treatment after 24 h in the cell cycle and death. **(A–D)** Bar graph showing the percentage of cells in each phase of the cell cycle in HL-60, THP-1, OCI-AML2, and OCI-AML3, respectively. **(E–H)** Representative histogram of the phases of the cell cycle in HL-60, THP-1, OCI-AML2, and OCI-AML3, respectively. The color red in the histogram represents the non-treatment controls, blue for nimesulide 100 μM of treatment, green for prednisolone 100 μM of treatment, and purple for nimesulide in combination with prednisolone 100 μM. Data shown are representative of 2 independent experiments and presented as mean ± SEM (n = 4). The p-value was determined using the two-way ANOVA multiple comparisons with Turkey’s correction, *p < 0.05; **p < 0.01; ****p < 0.001. Control: non-treated cells; DMSO: treated with DMSO (dilution vehicle); N100: nimesulide 100 µM; P100: prednisolone 100 µM; NP100: combination of nimesulide and prednisolone 100 µM; Cytarabine: treated with cytarabine 2.5 µM. DMSO, dimethyl sulfoxide. ns, not significant.

To investigate the effect of nimesulide and prednisolone in AML, we treated HL-60, THP-1, OCI-AML2, and OCI-AML3 cells lines with these drugs at 100 μM. To assess the effect on the cell cycle at 24 h, we evaluated the DNA content and fluorescence intensity in the sub-G0 (cell death), G0/G1, S, and G2/M phases (**Supplementary Figure 1**). We observed that treatment with nimesulide alone increased the number of cells in sub-G0 (dead cells) when compared to the control, and the combination of nimesulide with prednisolone causes a statistically significant increase in sub-G0 cells when compared to the treatment of nimesulide alone with the exception of cell line OCI-AML3 (**Figure 2**). On the contrary, isolated prednisolone treatment did not cause cell death or changes in the cell cycle (**Figure 2**). The OCI-AML3 cell line, which is also resistant to cytarabine, was less sensitive to treatment with anti-inflammatory drugs than the other cell lines. Overall, we found a significant induction of cell death at 24 h after the treatment with nimesulide alone and in combination with prednisolone.

**Figure 3:**
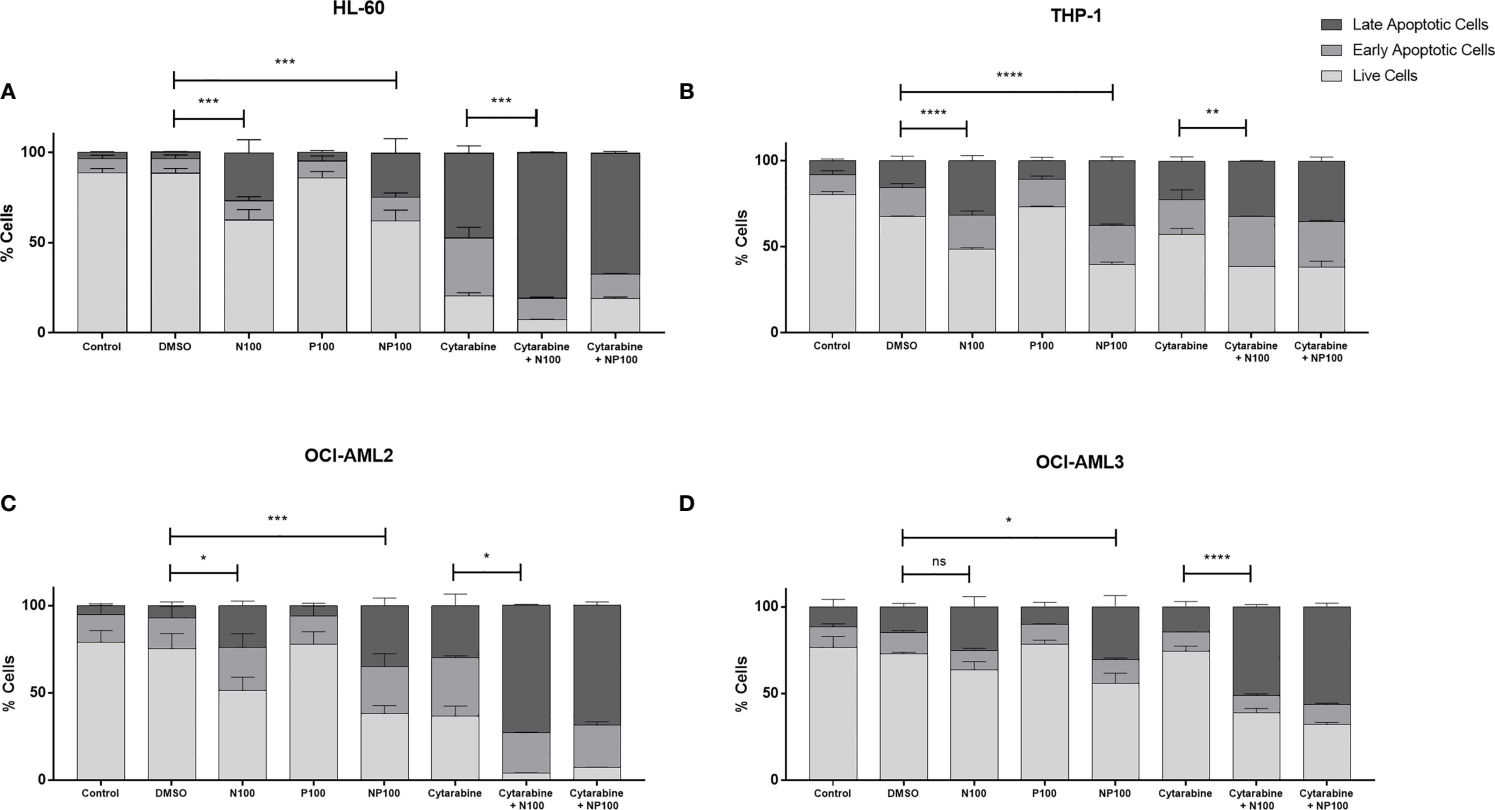
Pro-apoptotic effect of treatment with nimesulide, prednisolone, and cytarabine. Bar graph showing the percentage of apoptotic cells in each cell line. **(A)** HL-60, **(B)** THP-1, **(C)** OCI-AML2, and **(D)** OCI-AML3 after treatment with the drugs alone and in combination for 24 h. The data presented are representative of 2 independent experiments and presented as mean ± SEM (n = 5). The p-value was determined using the two-way ANOVA multiple comparisons with Turkey’s correction, *p < 0.05; **p < 0.01; ***p < 0.001; ****p < 0.001. Control: non-treated cells; DMSO: treated with DMSO (dilution vehicle); N100: nimesulide 100 µM; P100: prednisolone 100 µM; NP100: combination of nimesulide and prednisolone 100 µM; Cytarabine: treated with cytarabine 2.5 µM; Cytarabine+N100; combination of nimesulide 100 µM and cytarabine 2.5 µM; Cytarabine+NP100: combination of nimesulide and prednisolone 100 µM and cytarabine 2.5 µM. DMSO, dimethyl sulfoxide. ns, not significant.

To characterize the mechanism of cell death, the proportions of early and late apoptotic cells were evaluated by flow cytometry after treatments for 24 h (**Supplementary Figures 2**). We can observe in the cell lines a significant decrease in live cells when comparing controls with treatment with nimesulide alone, with the exception of OCI-AML3, which proved more resistant to treatment (**Figure 3**). Treatment with prednisolone alone did not induce apoptosis in cell lines; however, it increased the proportion of cells in late apoptosis in THP-1, OCI-AML2, and OCI-AML3 cell lines. Treatment with anti-inflammatory drugs did not induce a significant increase in early apoptotic cells; nevertheless, it causes more cell death than cytarabine in THP-1 and OCI-AML3. In addition, nimesulide enhanced the effect of cytarabine, a standard chemotherapy agent for the treatment of AML in cell death induction in all cell lines. In the cell lines more sensitive to cytarabine, HL-60, and OCI-AML2, the addition of nimesulide to the treatment led to the death of almost all leukemic cells (**Figures 3A, C**).

### Gene Co-Expression Network Analysis Reveals Mechanisms Associated With Anti-Leukemia Effect of Anti-Inflammatory Drugs

In order to find a common mechanism behind the induction of cell death in AML cell lines after treatment with nimesulide alone and in combination with prednisolone, we decided to perform whole-transcriptome sequencing for each condition. All samples had at least 30 million reads and a Phred Score above Q30. We then performed principal component analysis (PCA) on raw gene expression data, normalized by FPKM (**Supplementary Figure 3**) to evaluate the clustering behavior and to remove possible outliers. We considered four experimental treatment groups (DMSO, prednisolone, nimesulide, and prednisolone plus nimesulide) from two separate experiments (duplicates). We observed that OCI-AML3 clustered significantly distantly from the three other cell lines; therefore, we decided to exclude OCI-AML3 from subsequent analysis. PCA also showed that the samples were clustered by experiment. In order to avoid data distortion while trying to correct this batch effect, we processed and analyzed the data from the two experiments separately, using experiment 2 analysis as a validation of experiment 1.

**Figure 4:**
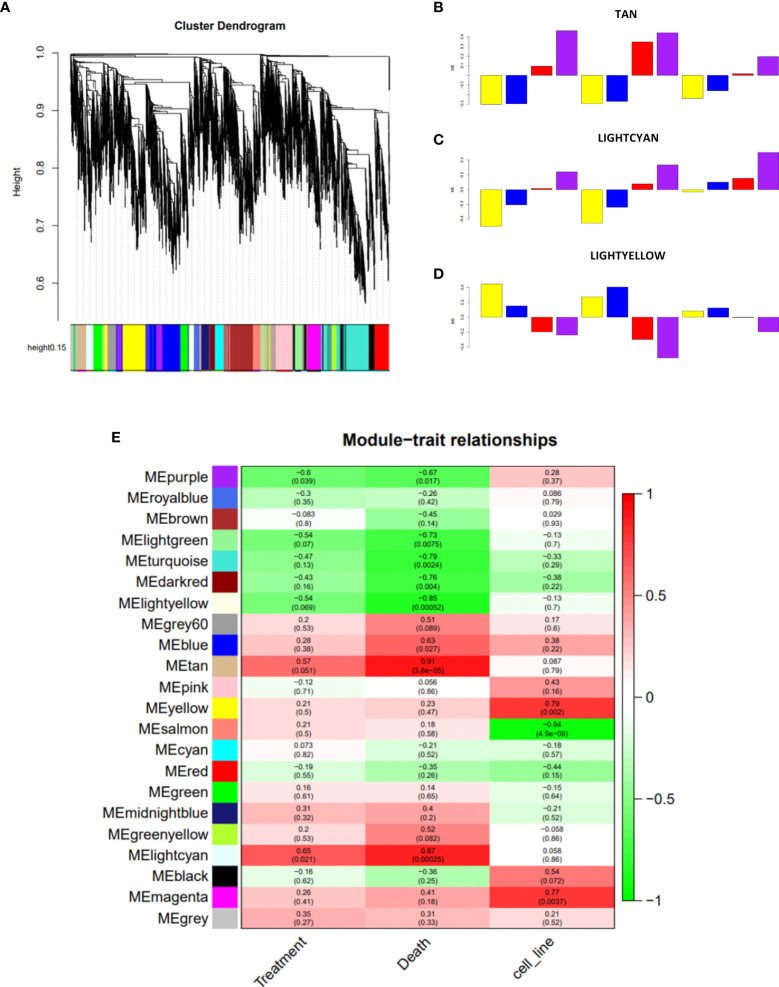
Construction of co-expression network of experiment 1. **(A)** Network analysis dendrogram showing clustering of genes based on topological overlap for identification of modules of co-regulated genes in experiment 1. **(B–D)** Bar plots of the samples representing their variation against the significant modules Tan, Lightcyan, and Lightyellow, respectively. The color of the bar is associated with the group: DMSO (yellow), treatment with prednisolone (blue), treatment with nimesulide (red), and treatment with nimesulide plus prednisolone (purple). The representation of cell lines in bar plots is in the order HL-60, THP-1, and OCI-AML2. **(E)** Module–trait relationships showing the sets of genes (modules) generated for experiment 1 and correlation of the detected modules with the variables treatment, death, and cell line. Each row represents a module eigengene; each column to a trait. Each cell contains the corresponding correlation and p-value. DMSO, dimethyl sulfoxide.

Due to the biological and genetic heterogeneities of AML cell lines, it is expected that they present dysregulation of different genes, which, however, probably participate in similar functional networks. In order to explore the data in a system-level context, we used WGCNA (18) analysis to assign genes with similar expression patterns into a module. The construction of the network and the gene assignment were obtained by selecting the ~10,000 genes with the greatest variation among the expressed genes, and then, the dendrogram representing the separation of the modules through colors was generated (**Figure 4A**). The gene expression levels for each generated module were then summarized by the first principal component (eigengene module) for each sample, and then we evaluated the correlation of the eigengene module with our variables of interest: treatment (treated vs. untreated cells), cell death level (DMSO vs. prednisolone vs. nimesulide vs. prednisolone plus nimesulide), and cell line (HL60, THP-1, and OCI-AML2). With this analysis, we identified which gene networks (eigengene modules), represented on the y-axis of **Figure 4E**, are correlated with cell lines, treatment, and death levels. The defined significance values were assigned by dividing p = 0.05 by the number of modules generated.

WGCNA of experiment 1 revealed twenty-two modules of co-expressed genes. We observed three modules that showed a significant correlation (p-adj ≤ 0.0022) with death levels: downregulated Lightyellow module (p = 0.00052), whose expression levels are inversely correlated with death levels, and upregulated modules Tan (p = 0.000038) and Lightcyan (p = 0.00025), which showed a positive correlation (**Figure 4E**). In addition to the correlation seen from the three modules with the levels of death, we can observe from the bar plots (**Figures 4B–D**) how the expression of each sample of the three cell lines behaves in the face of treatments. The figures show that samples treated with DMSO (yellow bar) and prednisolone (blue bar) are inversely correlated with nimesulide alone (red bar) and combined with prednisolone (purple bar) treatments. The drug combination has a stronger correlation with modules than nimesulide alone. The same WGCNA was performed for experiment 2, and modules were generated and correlated with the same variables (**Supplementary Figure 4**). The biological nature of these significant treatment-associated modules is supported by evidence of protein–protein interaction performed by the STRING platform (**Supplementary Figure 7**).

In order to find common genes identified in both experiments, the overlap between the significant modules from experiment 1 with the significant modules from experiment 2 was determined (**Supplementary Table 1**). We observed that the upregulated Tan module overlapped with the genes of the Turquoise, Red, and Salmon modules from experiment 2; the other upregulated Lightcyan module overlapped with the Turquoise, Red, and Yellow modules; and the downregulated Lightyellow module had overlap with Cyan and Grey60 modules. The number of total genes within each module of both experiments is described in **Supplementary Table 2**.

### Functional Enrichment Analysis of Modules

To explore the potential biological function of critical modules, we conducted a functional enrichment analysis. As shown in **Table 1**, the modules upregulated in experiment 1 (Lightcyan and Tan) were significantly enriched for genes related to the regulation of autophagy and apoptotic processes, and the module downregulated (Lightyellow) was significantly enriched for cell cycle and RNA splicing pathways genes. We found enrichment for these same upregulated and downregulated pathways in the modules of experiment 2 that overlapped with experiment 1, thus validating the results found in experiment 1 (**Supplementary Table 3**).

**Table 1:** Enriched pathways relevant to the significant modules of experiment 1.

Upregulated—Lightcyan			
Annotation cluster 1—enrichment score 2.73			
GOTERM_BP_FAT	No. genes	p-Value	Genes
GO:0097576: Vacuole fusion	9	5.1E−4	ATP6V1H, CLEC16A, GABARAPL1, NBR1, RAB24, VIPAS39, VPS33B, VPS39, VPS51, WDR24, WIPI2, ATG13, ATG14, ATG7, CALCOCO2, FAM160A2, FLCN, LGALS8, GAA, GOLGA2, IFT20, MCOLN1, MVB12A, MTMR14, PACS2, PRKAG1, SIRT2, SNAP23, SNAP29, SYT11, SYTL3, STX1A, STX5, TECPR1, TMEM127, TMEM175, TRIM5, TPP1, TSC1, USP30, ULK1, VAT1, ZFYVE1, ZBTB17, ZKSCAN3, ZKSCAN4
GO:0097352: Autophagosome maturation	8	1.1E−3	
GO:0048284: Organelle fusion	17	1.4E−3	
GO:0007033: Vacuole organization	22	2.5E−3	
GO:0006914: Autophagy	35	3.6E−3	
GO:0016236: Macroautophagy	21	6.1E−3	
**Upregulated—Tan**			
**Annotation cluster 11—enrichment score 1.75**			
**GOTERM_BP_FAT**	**No. genes**	**p-Value**	**Genes**
GO:0070059: Intrinsic apoptotic signaling pathway in response to endoplasmic reticulum stress	13	2.2E−4	BRCA1, CEBPB, CREBRF, CHAC1, DDIT3, DDIT4, EDEM1, ERCC6, FYN, JUN, RBCK1, WIPI1, XBP1, ATF3, ATF4, ASNS, ATXN3, CASP4, CTH, DPF2, ERN1, EEF2, EIF2AK3, FOXO3, GCLC, HERPUD1, IVNS1ABP, ITPR1, ITGA6, MAP2K5, MLH1, NRBF2, PMAIP1, PARP16, PPP1R15A, PPP2R5C, P2RX4, RHBDD1, RNF41, SESN2, SIAH1, SIRT1, STC2, TRIB3, TRIM39, TP63, TPT1, USP25
GO:0034976: Response to endoplasmic reticulum stress	29	1.9E−2	
GO:0097193: Intrinsic apoptotic signaling pathway	23	8.9E−2	
GO:0097190: Apoptotic signaling pathway	36	2.6E−1	
**Downregulated—Lightyellow**			
**Annotation cluster 1—enrichment score 5.87**			
**GOTERM_BP_FAT**	**No. genes**	**p-Value**	**Genes**
GO:0006397: mRNA processing	49	4.8E−8	BMS1, CNOT11, DCAF13, FASTKD5, DDX1, GAR1, RBM25, DBR1, DDX46, EIF4A3, HNRNPU, CCAR1, UBL5, SNRPD1, MAGOH, ZNF326, SNRPD3, TXNL4A, RBM17, THOC3, RBMXL1, SARNP, FASTKD5, STRAP, PPIH, SNRPE, SNRPF, HNRNPH3, AURKAIP1, SNRPC, LUC7L2, SRSF9, SF3B4, SLBP, U2AF1, CCNB1, POLR2B, POLR2C, LEO1, SAP18, POLR2K, HNRNPA0, LSM1, CWC15, LSM3, HNRNPM, SNRNP40, LSM6, PHF5A, HNRNPK, HNRNPC, TARDBP, HSPA1B, HSPA1A, THUMPD1, TSR3, DBR1, EXOSC3, EXOSC9, MTFMT, PIN4, RRP36, RPS27L, SLBP, TRMT10C, TGFB1, ZC3HAV1, ZBTB8OS
GO:0008380: RNA splicing	44	1.2E−7	
GO:0000375: RNA splicing *via* transesterification reactions	37	1.5E−7	
GO:0000377: RNA splicing *via* transesterification reactions with bulged adenosine	36	3.5E−7	
GO:0000398: mRNA splicing *via* spliceosome	36	3.5E−7	
GO:0016071: mRNA metabolic process	53	7.4E−5	
GO:0006396: RNA processing	63	9.9E−4	
**Annotation cluster 10—enrichment score 3.9**			
**GOTERM_BP_FAT**	**No. genes**	**p-Value**	**Genes**
GO:1901990: Regulation of mitotic cell cycle phase transition	33	9.3E−6	BUB1, BUB3, CNOT11, NAE1, NEK6, RAD1, RINT1, SKP1, TRIAP1, AURKAIP1, BIRC5, CALM2, CUL3, CCNB1, FEN1, HDAC8, NUSAP1, PIK3R4, PCNA, PSMC2, PSMC3, PSMD1, PSMD10, PSMD11, PSMD6, PSMD7, PSMD9, PSME3, PSMA2, PSMA3, PSMA4, PSMA5, PSMA6, PSMB1, PSMB2, PSMB5, RPA2, RPS27L, RNF4, TGFB1, UBE2C
GO:1901987: Regulation of cell cycle phase transition	33	2.1E−5	
GO:0010564: Regulation of cell cycle process	41	5.7E−4	
GO:0007346: Regulation of mitotic cell cycle	35	2.3E−3	

### A Subset of Genes Upregulated *In Vitro* Were Also Upregulated in the Patient’s Bone Marrow After Nimesulide Treatment

In order to evaluate whether the genes identified in the eigengene modules in the cell line experiments followed the same expression trend among the patient’s transcriptomes before and after nimesulide treatment, we adopted the following approach. Since it was not possible to generate a statistical analysis of the patient’s data as we had only one transcriptome sample for each treatment time (a total of three transcriptomes), we created two gene expression ratios based on the FPKM ratio for each gene. Ratio 1 was FPKM from D+21 after nimesulide treatment (early) divided by FPKM at diagnosis, and ratio 2 was FPKM from D+63 after nimesulide treatment (late) divided by FPKM at diagnosis. Then, both ratios were ranked from the highest to lowest in order to show at the top of the rank the genes with the highest fold-change and at the bottom of the rank the genes with the lowest fold-change (including downregulated genes). Afterward, we performed an enrichment test to see if the genes from the modules of experiment 1 (Lightyellow, Tan, and Lightcyan) generated by the WGCNA had a non-random distribution along with this ranked list, that is, if upregulated genes tended to occur more on the top or the bottom of the ranks and vice versa.

We verified that when comparing the patient’s early rank with the genes from modules from experiment 1, related to autophagy (Lightcyan) and RNA splicing and cell cycle (Lightyellow), those genes presented a non-random distribution, being concentrated among the genes with the highest fold-change, with p-values of 0.003 and 0.004, respectively (**Table 2**). While the expression direction of genes related to autophagy was coherent between cell line and patient transcriptome ratio (upregulated expression), genes related to RNA splicing were not. Therefore, genes associated with autophagy were upregulated after nimesulide treatment both in the patient’s transcriptome and in cell line experiments.

**Table 2:** Correlation of the patient’s ranked genes with modules of experiment 1.

Correlation	Number of genes	Expression	p-Value
Tan–Early	746	Up	0.413476285050312
Tan–Late	752	Up	0.326814219505457
Lightcyan–Early	618	Up	0.00366376723337888
Lightcyan–Late	618	Down	0.960253266351426
Lightyellow–Early	478	Up	0.00462018230578614
Lightyellow–Late	482	Down	0.653726520709975

We have also evaluated changes in gene expression induced by nimesulide in mutated driver genes in the cell lines. The change in expression of DNMT3A was heterogeneous. While it was slightly reduced in THP1, it was marginally increased in HL60 and OCI-AML3 and significantly increased in OCI-AML3 (**Supplementary Figure 5**). The changes in gene expression of mutated alleles in the cell line OCI-AML3 were not significant (**Supplementary Figure 6**).

### Nimesulide Potentiates the Effect of Drugs Approved for Acute Myeloid Leukemia Treatment

**Figure 5:**
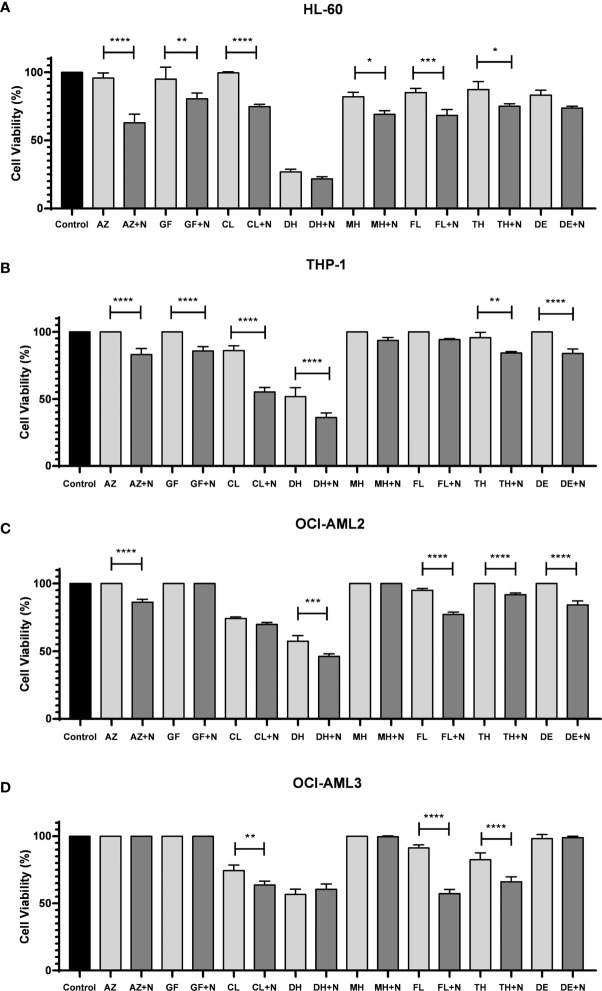
Cell viability assay after treatment with selected FDA-approved anti-AML drugs combined with nimesulide. **(A–D)** Bar graph showing cell viability on cell lines (HL-60, THP-1, OCI-AML2, and OCI-AML3) after being treated and incubated for 24 h. Data shown are representative of an independent experiment in triplicate and presented as mean ± SD (n = 3). The p-value was determined using the one-way ANOVA comparing the drug alone and in combination with nimesulide, *p < 0.05; **p < 0.01; ***p < 0.001; ****p < 0.0001. Control: DMSO. N, nimesulide; AZ, azacitidine; GF, gilteritinib fumarate; CL, cladribine; DH, doxorubicin hydrochloride; MH, mitoxantrone hydrochloride; FL, fludarabine; TH, thioguanine; DE, decitabine; FDA, Food and Drug Administration; AML, acute myeloid leukemia; DMSO, dimethyl sulfoxide.

To further explore the potential of nimesulide in combination with other approved AML treatments, we evaluated the cytotoxic effects of the following drugs alone and in combination with nimesulide: azacitidine, gilteritinib fumarate, cladribine, doxorubicin hydrochloride, mitoxantrone hydrochloride, fludarabine, thioguanine, and decitabine. Nimesulide potentiates the effect of all chosen AML treatment drugs, decreasing the viability of leukemic cells by different proportions depending on the type of cell line (**Figure 5**). We consider that nimesulide potentiated the drug’s action when after its addition there was twice as much death as compared to the drug alone. We observed that nimesulide potentiated the effect of the following drugs: azacitidine, gilteritinib fumarate, cladribine, and fludarabine in the HL-60 cell line; azacitidine, gilteritinib fumarate, cladribine, thioguanine, and decitabine in the THP-1 cell line; azacitidine, fludarabine, thioguanine, and decitabine in the OCI-AML2 cell line; and fludarabine in the OCI-AML3 cell line.

## Discussion

In the present work, we reported the first-in-human AML partial remission induced by nimesulide. The response was initially characterized by the acquisition of myeloid lineage markers CD11b, CD13, and CD15 and decreased expression of progenitor antigens, HLA-DR, and CD117 with the posterior reduction in blast count characterizing a partial response. While the patient presented an 82% reduction in bone marrow blast cell counts and complete blast clearance in peripheral blood, serial whole-exome sequencing revealed stability of variant allele frequencies, compatible with differentiation of leukemic blasts. Although rare, partial and complete remissions have been previously described in untreated AML and myelodysplastic syndrome (MDS) patients (19, 20). While the mechanisms involved in such remissions are unknown, there has been speculation about a possible immune-mediated anti-leukemic effect, since an episode of infection is commonly associated with the responses (19). We believe this was not the case in our patient, since there was no infection associated and blast differentiation has not been described and is neither expected, as a result of the immune-mediated anti-leukemic effect. An anti-leukemic effect of nimesulide has been previously demonstrated *in vitro* (11, 12) and a xenograft model (13), suggesting that this compound may become clinically relevant for AML treatment. We recognize that the duration of the response was short, lasting approximately 3 months, but it is worth mentioning that even venetoclax and midostaurin, both FDA-approved therapies for AML, showed limited efficacy with short duration responses as single agents (21, 22), making the combination with other drugs necessary.

We have also confirmed the previously described *in vitro* induction of cell death and cell cycle arrest in AML cell lines treated with COX-2 inhibitors, including nimesulide (11, 12), and we characterized the biological mechanisms associated with this effect. Since our patient was using prednisolone when nimesulide was started, we included samples treated with prednisolone only, nimesulide only, and the combination in our experimental design to evaluate a possible synergistic effect. Even though there are findings suggesting a role for glucocorticoids in the treatment of AML in specific settings (23, 24), we could not detect an anti-leukemic activity in our experiments with prednisolone alone, but it potentiated the pro-apoptotic and cell cycle effects of nimesulide in three out of four cell lines tested, suggesting that the combination of nimesulide and prednisolone may have had a role in the observed partial response. We could not demonstrate the previously described *in vitro* differentiation of leukemic blasts induced by COX inhibitors, including nimesulide (11, 12), since DMSO alone induced the expression of myeloid differentiation markers (data not shown). We have confirmed the previously described MYC downregulation induced by COX-inhibitors in AML (12) and revealed novel mechanisms. The most significant changes induced by treatment were upregulation of genes associated with apoptosis and autophagy and downregulation of cell cycle and RNA splicing-associated genes.

To further understand if these cellular processes identified in the *in vitro* studies were also operating in the patient’s cells, we evaluated gene expression changes associated with *in vivo* partial response induced by nimesulide treatment. Genes associated with autophagy were also upregulated in the patient’s cell after treatment with nimesulide, confirming the *in vitro* findings. Autophagy has context-dependent roles in cancer, and interventions to stimulate or inhibit autophagy have been proposed as cancer therapies (25–27), including AML (28), and tested in clinical trials for different tumor types (29–32). In addition, it has been previously shown that autophagy plays a crucial role in hematopoietic differentiation at several stages, including myeloid differentiation (33). Further studies are necessary to better characterize the role of autophagy in cell death induction mediated by the use of nimesulide. Another commonality between *in vitro* and *in vivo* findings was the upregulation of DNMT3A after treatment with nimesulide both in the patient sample and in the OCI3-AML cell line (**Figure 1D** and **Supplementary Figure 5**). Of note, OCI-AML3 was the closest cell line in terms of gene mutations when compared to the patient, since both have mutations in DNMT3A, NPM1, and NRAS. It is possible that DNMT3A may have a role in the response to nimesulide observed in our patients since it has been shown that DNMT3A is essential for hematopoietic differentiation (34). Nevertheless, heterozygous mutation, as observed in AML, including our patient, normally does not affect differentiation and has no phenotype in animal models (35). Further studies are necessary to clarify the role of DNMT3A in the response to nimesulide.

Finally, we recognize that nimesulide is unlikely to benefit AML patients as a single agent; therefore, we explored the potential of combining nimesulide with established anti-AML therapies. Nimesulide potentiates the effect of drugs already approved for AML, suggesting that nimesulide may have a role in combination with standard AML treatment.

In conclusion, we have described the first-in-human AML partial response induced by nimesulide and demonstrated upregulation of autophagy associated genes after treatment with nimesulide both *in vitro* and *in vivo*. In addition, we have shown that *in vitro* combination of nimesulide with anti-AML FDA-approved therapies increases cell death, suggesting a potential for the utilization of this drug in AML treatment.

## Data Availability Statement

The datasets presented in this study can be found in online repositories. The names of the repository/repositories and accession number(s) can be found below: NCBI's Sequence Read Archive (SRA) with BioProject ID: PRJNA817613 (https://www.ncbi.nlm.nih.gov/bioproject/PRJNA817613).

## Ethics Statement

The studies involving human participants were reviewed and approved by Plataforma Brasil. The patients/participants provided their written informed consent to participate in this study. CAAE 50594315.7.1001.007 and CAAE 19826619.60000.0071.

## Author Contributions

All authors had direct contributions to the elaboration of this manuscript. The first author, VT, contributed to the performance of the *in vitro* experiments, preparation of samples for sequencing, analysis of cellular and bioinformatics data, and writing of this manuscript. RP performed the raw sequencing data alignment and the recall of variants of the patient samples and performed with KG-O the WGCNA. BC performed the drug screening experiment. The FS and NH contributed to the review of the bioinformatics and clinical data analysis, respectively. PC contributed to the creation of the experimental design of the project and critical analysis of the results and writing of this manuscript. All authors listed have made a substantial, direct, and intellectual contribution to the work and approved it for publication.

## Funding

This work was supported by grants from Programa Nacional de Apoio à Atenção Oncológica (PRONON) and The Applebaum Foundation.

## Conflict of Interest

Author RP was employed by company Grupo Pardini.

The remaining authors declare that the research was conducted in the absence of any commercial or financial relationships that could be construed as a potential conflict of interest.

## Publisher’s Note

All claims expressed in this article are solely those of the authors and do not necessarily represent those of their affiliated organizations, or those of the publisher, the editors and the reviewers. Any product that may be evaluated in this article, or claim that may be made by its manufacturer, is not guaranteed or endorsed by the publisher.
