# Supplementary material for: Molecular Characterization of a First-in-Human Clinical Response to Nimesulide in Acute Myeloid Leukemia
Source: Front Oncol. 2022 Jun 8;12:874168. doi: 10.3389/fonc.2022.874168 (PMC9215211; doi:10.3389/fonc.2022.874168)
Supplement: Supplementary file 1 [file DataSheet_1.docx]

Supplementary Material

# Supplementary Materials and Methods

**1.1. Whole Exome Sequencing**

The DNA of the patient's pre- and post-treatment samples extracted with DNeasy Mini Kit (Qiagen), following manufacturer's instructions, were analyzed through either whole exome sequencing (WES). Control DNA for WES was extracted from a paired normal skin biopsy, and exons were enriched using SureSelect Human All Exon V4 (Agilent Technologies). For the custom panel the target regions were enriched using the Custom SureSelect hybrid capture system (Agilent Technologies). Prepared libraries were sequenced on a Illumina HiSeq 2000. Library preparation and sequencing were done at Admera Health Biopharma Services in New York, NY and analysis was done was at Hospital Israelita Albert Einstein (HIAE). The bioinformatics pipeline of detection of driver genetic abnormalities was used as described previously.1

**1.2. RNA extraction**

RNA was extracted with RNeasy Mini Kit (Qiagen) following manufacturer's instructions. Briefly, cells lines were inoculated at a density of 1x106/mL into a 24-well culture plate and after treatment with the drugs for 4 hours, RNA was extracted. Purified RNA was eluted in ultra-pure water, free of endonucleases, quantified by NanoDrop One / One Microvolume UV-Vis Spectrophotometer (ThermoFisher), taking into account the absorbances ratios 260/280 and 260/230 nm. The quality and integrity was checked on RNA ScreenTape® (Agilent Technologies) to measure the RNA integrity number (RIN). The same protocol was used to extract RNA from three samples of the patient's bone marrow at three different times: at diagnosis and at two different times after nimesulide.

**1.3. Library Preparation and Sequencing**

Library preparation and sequencing were performed at the Oklahoma Medical Research Foundation Genomics Core (USA). Briefly, for library preparation, NEBNext® Ultra II Directional RNA Library Prep Kit for Illumina® was used, following the protocol using Poly(A) mRNA Magnetic Isolation Module. For RNA sequencing, approximately 500 ng, with high quality RNA samples (RIN > 7), of total RNA from each experimental group were converted into their respective cDNA library. The NEBNext Poly(A) mRNA Magnetic Isolation Module (Illumina) use oligo d(T) beads, which bind to the poly(A) tail of eukaryotic mRNA will be enriched. After generation of Double-stranded cDNA, the products reactions were purified by NEBNext Sample Purification Beads and the DNA ends were repaired using NEBNext End Prep Enzyme, followed by NEBNext adaptor for Illumina ligation using Blunt/TA Ligase master mix. After another round of purification, adaptor-ligated DNA was amplified using using Ultra II Q5 Master Mix in the presence of NEBNext oligos. In the end, the PCR-enriched libraries were analyzed by a Bioanalyzer High Sensitivity DNA chip. Equimolar amounts of libraries were pooled and sequenced using an Illumina NextSeq 500, following the manufacturer's instructions.

**1.4. Bioinformatics pipeline**

The sequences obtained with the RNA-Seq technique were aligned in the human genome of reference GRCh37.75 by the software Spliced Transcripts Alignment to a Reference (STAR) v2.5 (http://github.com/alexdobin/STAR). After alignment, the RNA-Seq by Expectation-Maximization (RSEM) v1.3.0 software (https://github.com/deweylab/RSEM) was used to obtain a table with the FPKM expression of the mapped genes and filter readings of low quality.

**1.5. Quality analysis and removal of outliers**

An analysis of the overall expression profile of the samples was performed by means of clustering and principal component analysis in order to identify samples with discrepant expression profiles or clear batch effects and to remove the "outliers". Using the table with the raw FPKM data for each sequenced sample as input, PCA graphs were generated, grouping the data according to cell line, type of treatment and by replicates (experiment 1 and 2).

**1.6. Sequencing of leukemic cell lines**

DNA extraction from cell lines was performed using the DNeasy Mini kit (Qiagen), following the manufacturer's instructions. The extracted genomic DNAs were diluted to a concentration of 5ng/uL and submitted to the TruSight Myeloid Sequencing Panel protocol (Illumina). The panel covers the genes: ABL1, ASXL1, ATRX, BCOR, BCORL1, BRAF, CALR, CBL, CBLB, CBLC, CDKN2A, CEBPA, CSF3R, CUX1, DNMT3A, ETV6/TEL, EZH2, FBXW7, FLT3, GATA1, GATA2, GNAS, HRAS, IDH1, IDH2, IKZF1, JAK2, JAK3, KDM6A, KIT, KRAS, MLL, MPL, MYD88, NOTCH1, NPM1, NRAS, PDGFRA, PHF6, PTEN, PTPN11, RAD21, RUNX1, SETBP1, SF3B1, SMC1A, SMC3, SRSF2, STAG2, TET2, TP53, U2AF1, WT1 and ZRSR2. After the libraries were prepared, they were quantified by the Qubit dsDNA HS kit (Invitrogen) and the concentration in ng/ul was converted to nM. Then the library was denatured to 20pM and diluted to 1.8pM. All sequencing analyzes were performed on the Illumina NextSeq 500 instrument according to the manufacturer's instructions.

**1.7. Calling Somatic Variants**

The quality of the sequences was assessed using FastQC (https://www.bioinformatics.babraham.ac.uk/projects/fastqc/). The Fastq raw files were obtained after demultiplexing by the BCL2FASTq program. Then, the sequences were aligned to the human reference genome (version GRCh37 http://www.ensembl.org/Homo_sapiens/Info/Index) using BWA software version 0.7.10 (http://sourceforge.net/projects/ bio-bwa/). To mark PCR duplicates, Picard software, version 1.94 (http://picard.sourceforge.net) was used. For realignment around insertions/deletions and recalibration of the quality of the bases, the Genome Analysis Toolkit (GATK; version 2.7-4; http://www.broadinstitute.org/gatk/index.php) was used. The variant callers used were the MuTect1 and 2 software (http://www.broadinstitute.org/cancer/cga/mutect) to identify Single Nucleotide Variants (SNVs) somatic mutations; Pindel 0.2.4 software (http://gmt.genome.wustl.edu/pindel/0.2.4/index.html) for identification of deletions, insertions and somatic inversions; and Freebayes software to detect short sequence variants. Variant annotation was performed using the ANNOVAR software (http://www.openbioinformatics.org/annovar/). The IGV program (Integrative Genomics Viewer – Broad institute) was used to visualize the alignments through the BAM file.

**Supplementary Reference:**

# Santos FPS, Getta B, Masarova L, Famulare C, Schulman J, Datoguia TS, et al. Prognostic impact of RAS-pathway mutations in patients with myelofibrosis. Leukemia (2020) 34(3):799-810. doi:10.1038/s41375-019-0603-9

# Supplementary Figures and Tables

**Supplementary Figure 1. Effect of the anti-inflammatory drugs nimesulide and prednisolone on the cell cycle.** Histograms representing the distribution of DNA content in cell cycle phases after treatment with nimesulide, prednisiolone and citarabine on the cell lines HL-60, THP-1, OCI-AML2 and OCI-AML3 after 24 hours. Control: non-treated cells; DMSO: treated with DMSO (dilution vehicle); N100: nimesulide 100 µM; P100: prednisolone 100 µM; NP100: combination of nimesulide and prednisolone 100 µM; Cytarabine: treated with cytarabine 2.5 µM.


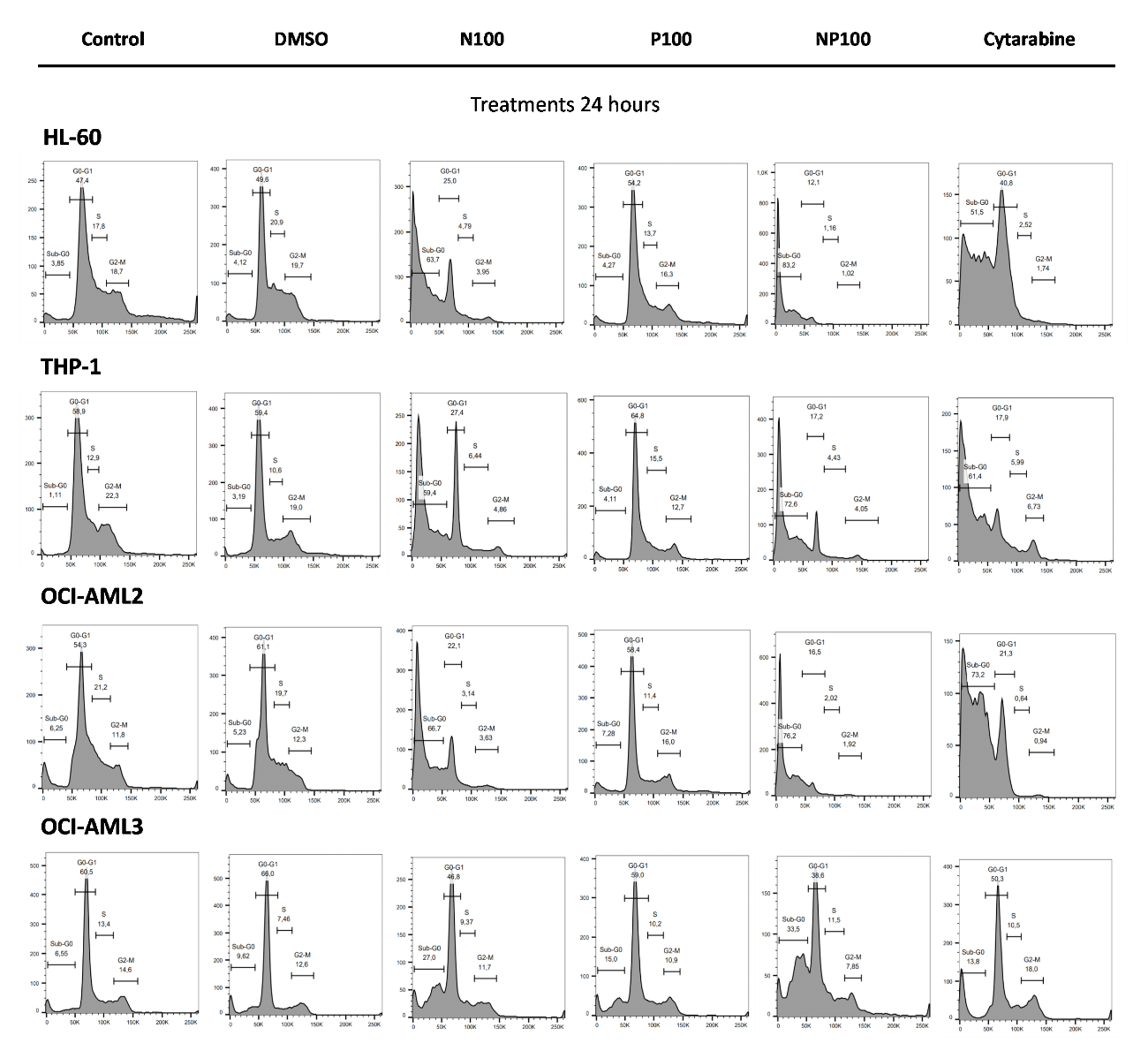


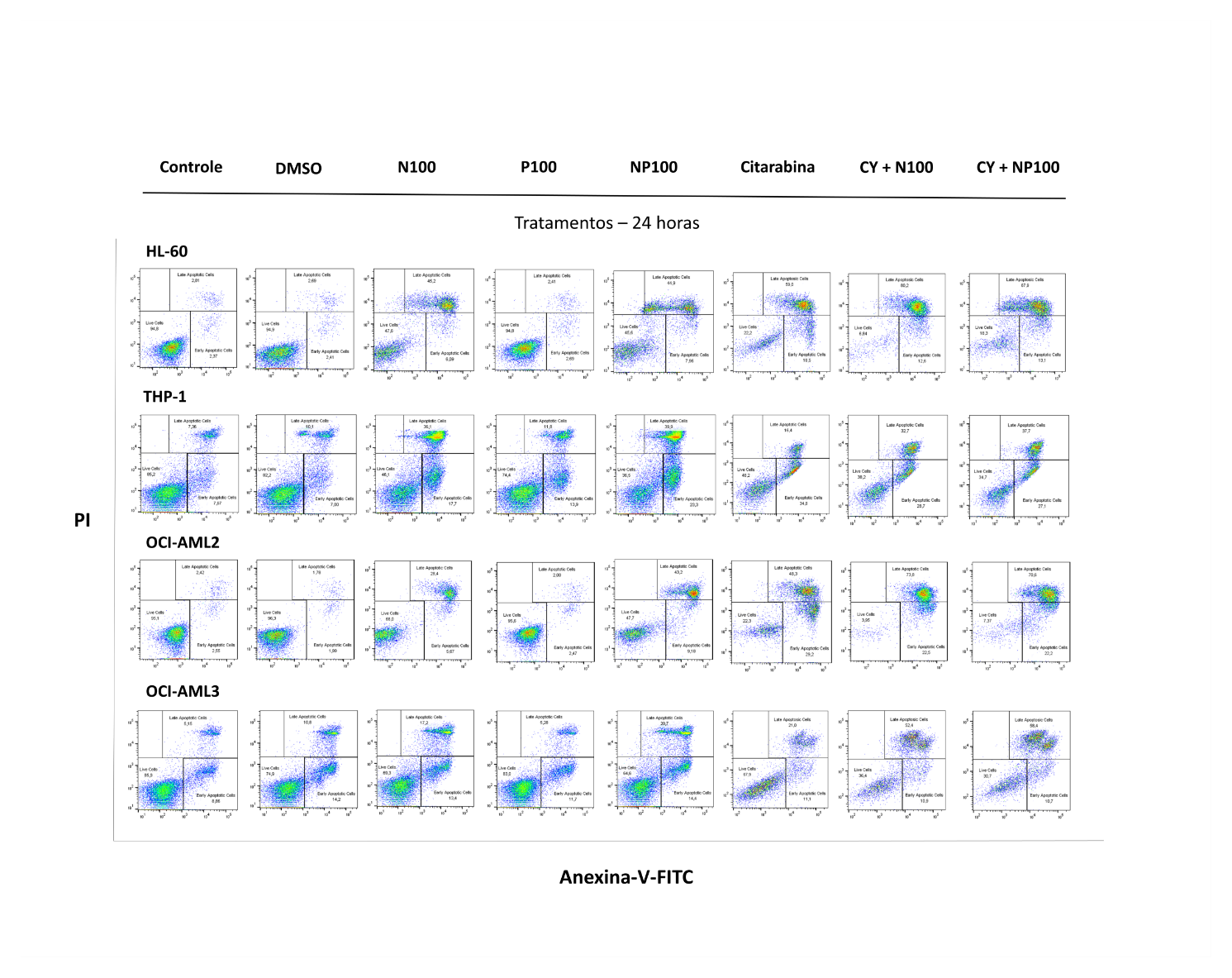
**Supplementary Figure 2. Apoptosis effect of anti-inflamatory drugs on leukemic cells lines.** Evaluation of proportions of early apoptotic cells (annexin V-FITC + / PI -) and late apoptotic cells (annexin V-FITC + / PI +) by flow cytometry after incubation of cells line HL-60, THP-1, OCIAML2 and OCI-AML3 with the following treatments. Control: non-treated cells; DMSO: treated with DMSO (dilution vehicle); N100: nimesulide 100 µM; P100: prednisolone 100 µM; NP100: combination of nimesulide and prednisolone 100 µM; Cytarabine: treated with cytarabine 2.5 µM; CY + N100: combination of nimesulide 100 µM and cytarabine 2.5 µM; CY + NP100: combination of nimesulide and prednisolone 100 µM and cytarabine 2.5 µM.

**Supplementary Figure 3. Principal component analysis of transcriptome samples.** Clustering of samples from experiment 1 by cell lines and treatment and experiment 2 by cell line, experiment, treatment and sequencing. DMSO: control; N100: treatment with nimesulide 100 μM; P100: treatment with prednisolone 100 μM; NP100: treatment with the combination of nimesulide and prednisolone.


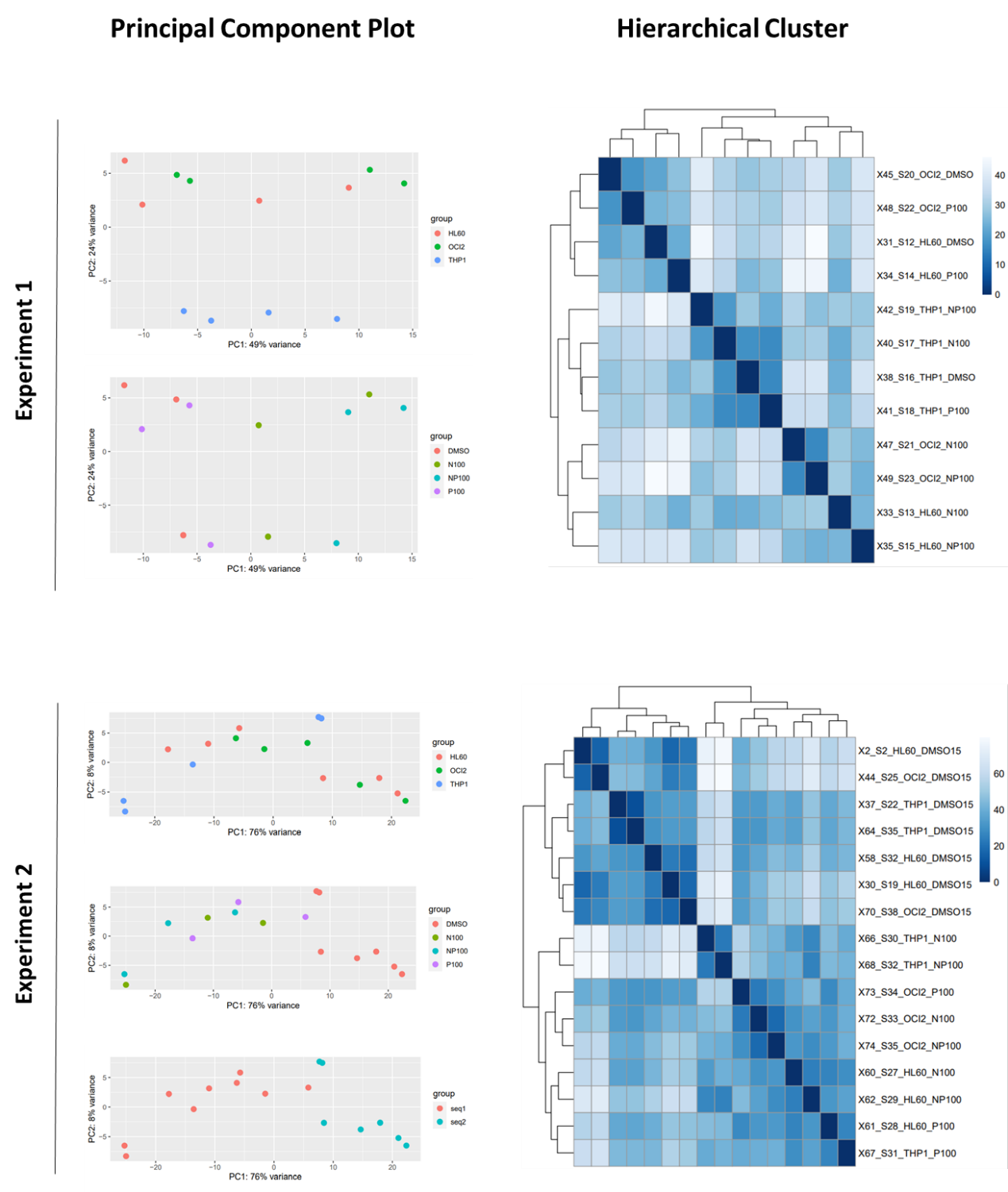


**Supplementary Figure 4. Construction of co-expression network of experiment 2.** (A) Network analysis dendrogram showing clustering of genes based on topological overlap for identification of modules of co-regulated genes in experiment 2. (B) Module-treait relationships showing the sets of genes (modules) generated for experiment 2 and correlation of the detected modules with the variables treatment, death and cell line. (C-J) Barplots of the samples representing their variation against the significant modules Salmon, Turquoise, Yellow, Red, Grey60, Greenyellow Cyan and Blue, respectively. The color of the bar is associated with the group: DMSO (yellow), treatment with prednisolone (blue), treatment with nimesulide (red) and treatment with nimesulide plus prednisolone (purple). The representation of cell lines in barplots is in the order HL-60, THP-1 and OCI-AML2, respectively.

**
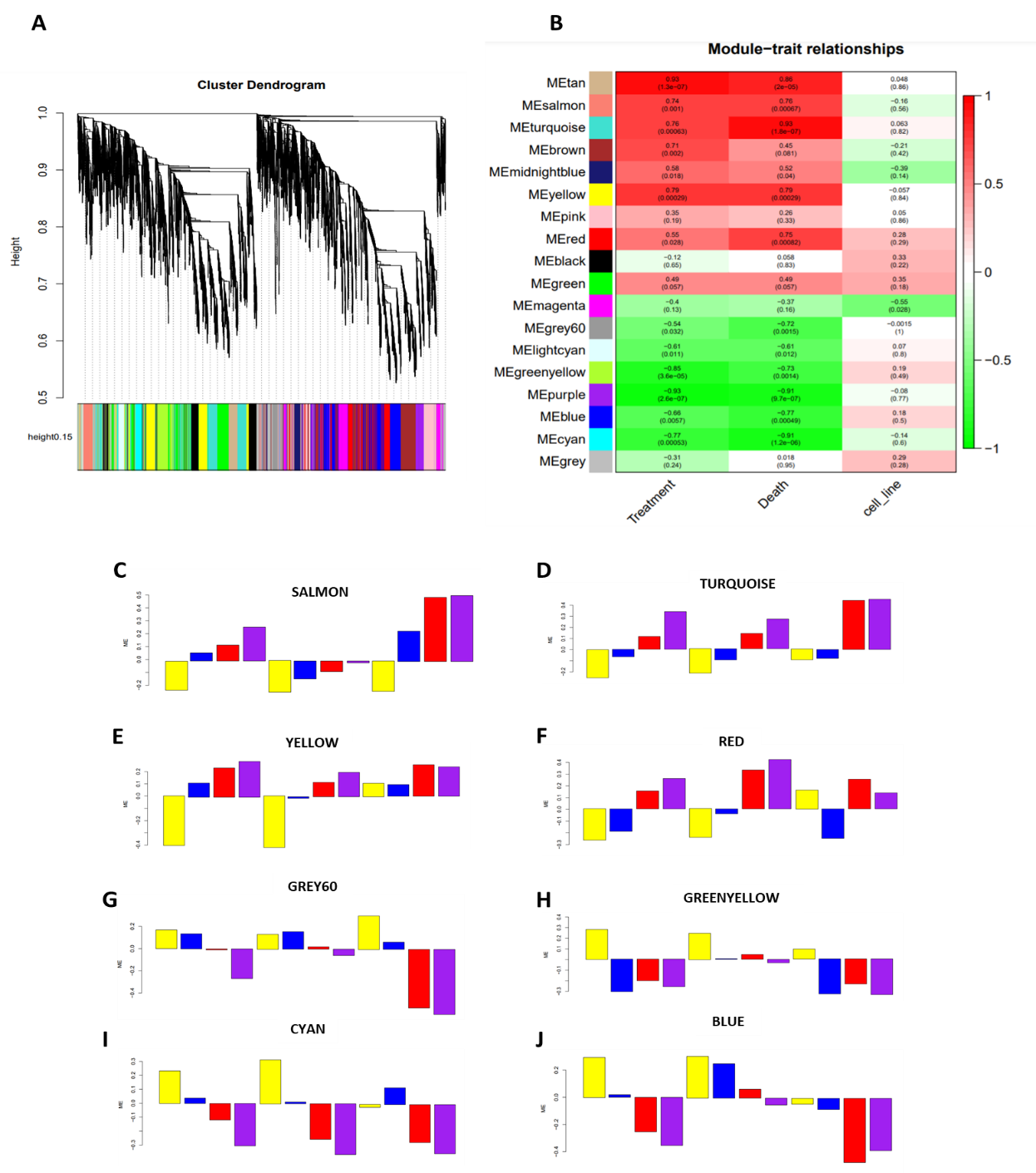
**

**Supplementary Figure 5. Cell lines DNMT3A gene expression changes after treatment with nimesulide.** Changes in DNMT3A gene expression in the cell lines HL-60, THP-1, OCI-AML2 and OCI-AML3 after 4 hours of treatment with DMSO (dilution vehicle) and nimesulide 100 µM.


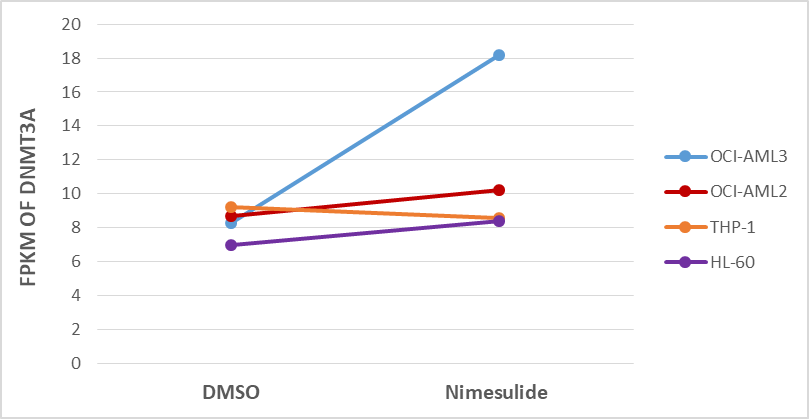


**Supplementary Figure 6. Allelic frequency of mutations found in the OCI-AML3 cell line in RNA samples.** Allelic frequency of DNMT3A, NP1M and NRAS mutations in transcriptome samples of the OCI-AML3 cell line after 4 hours of treatment with DMSO (dilution vehicle) and nimesulide 100 µM.

**
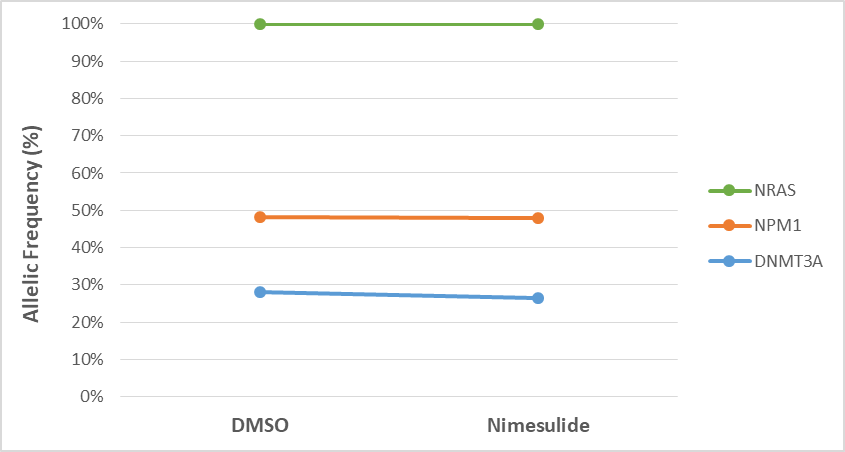
**

**Supplementary Figure 7. Protein-protein interaction networks showing biological evidence of interaction of genes assigned to each of the significant modules identified by WGCNA that presented overlap.** (A-K) Protein interaction of genes from modules (A) Tan, (B) Lightcyan, (C) Salmon, (D) Turquoise, (E) Yellow, (F) Red, (G) Lightyellow, (H) Grey60 and (I) Cyan.


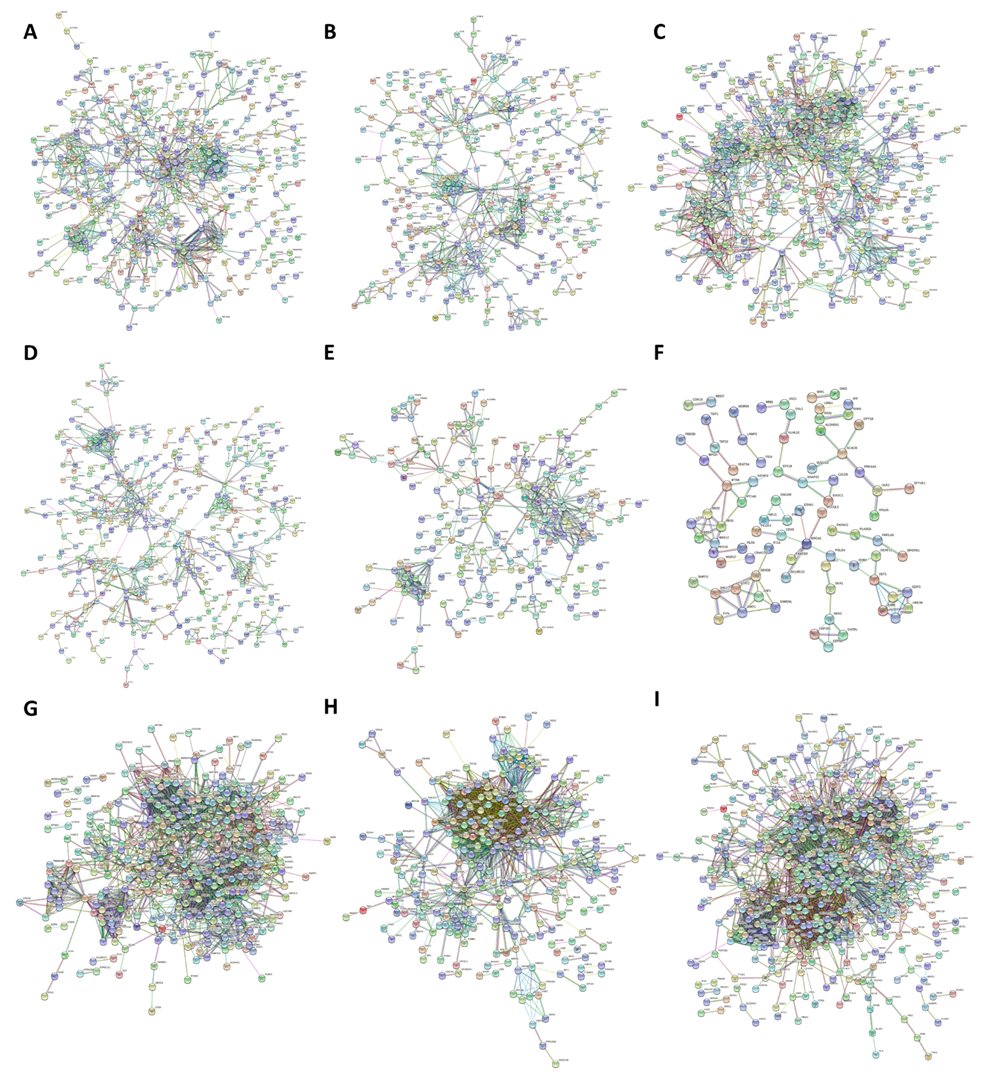


**Supplementary Table 1.** Overlap with the eigengenes modules of both experiments.

| **Module experiment 1** | **Module experimente 2** | **P value** | **Overlap genes** |
| --- | --- | --- | --- |
| **Tan** | Turquoise | 9,92E-132 | 287 |
|  | Salmon | 7,08E-13 | 122 |
|  | Red | 1,69E-11 | 69 |
| **Lightyellow** | Cyan | 4,67E-53 | 145 |
|  | Grey60 | 9,65E-07 | 58 |
| **Lightcyan** | Yellow | 2,32E-25 | 110 |
|  | Red | 1,68E-18 | 73 |
|  | Turquoise | 5,58E-16 | 115 |

**Supplementary Table 2.** Number of genes in co-expression module of experiment

**Supplementary Table 3.** Enriched pathways relevant to the modules of experiment 2 that overlapped with the modules of experiment 1.

| **Up-Regulated - Salmon** | | | |
| --- | --- | --- | --- |
| **Annotation Cluster 6 - Enrichment Score: 3.5** | | | |
| **GOTERM_BP_FAT** | **Nº Genes** | **P value** | **Genes** |
| GO:0008219: Cell death | 115 | 1.5E-4 | ADAM17, ADAM8, ADNP2, AKT1S1, BCL10, MCL1, BNIP1, BCL2L11, BCL2A1, BTG1, BTG2, DNAJC10, FBXO7, FOSL1, FOSL2, JUN, NFKBIA, NFKBID, PIM3, RAD18, REL, RELA, RYBP, RRN3, RABGGTB, SH3GLB1, SNW1, SRPK2, THOC1, TNFAIP3, TRAF1, TNFRSF10, TYRO3, WRN, WNT9A, ATF4, ATF6, ADNP, ADM, AIMP1, ANXA1, AHR, ATN1, BIRC3, CHST11, CSNK2A1, CSNK2A2, CTSL, F3, CTTN, CDKN1A, CSNK2A2, CSRNP1, DAP3, EGR1, EGR3, EMP1, FNTA, FLT3LG, GADD45A, HSPA9, HERPUD1, IER3, ING3, ITPR1, IP6K2, ITGA6, ITGB1, ITGB3BP, ICAM1, IL1B, ITCH, KLHL20, LMNA, MMP9, MAP2K7, MAP3K7, MEF2D, NSMAF, NFE2L2, NR4A1, NPM1, OXR1, PYGL, PHLDA1, PHLDA2, PLK3, PARP2, PRNP, PPP1R15A, RIPK2, RGCC, RTN4, RARA, RSL1D1, STK17B, STK40, SERPINE1, SIAH2, SIRT2, SLC25A6, SUSD6, STXBP1, TGFBR2, TMEM214, TNFSF10, TNF, TP63,, UBE2B, USP53, VAV3, VDAC2, ZNF16, ZNF304 |
| GO:0012501: Programmed cell death | 108 | 4.2E-4 |  |
| GO:0006915: Apoptotic process | 103 | 5.1E-4 |  |
| **Up-Regulated - Yellow** | | | |
| **Annotation Cluster 15 - Enrichment Score: 1.3** | | | |
| **GOTERM_BP_FAT** | **Nº Genes** | **P value** | **Genes** |
| GO:0006914: Autophagy | 28 | 1.0E-2 | BECN1, MTMR14, VPS4B, VPS26A, PSEN1, HDAC6, SCFD1, MAP1LC3B, MAPK8, MFN1, ATP6V0A2, HMOX1, USP30, SNAP29, SNX6, GABARAPL2, BNIP3L, WIPI2, ATG13, SIRT1, RAB33B, TMEM59, VAMP7, NEDD4, CHMP3, STAM2, TECPR2, RNF185, KAT8 |
| GO:0016236: Macroautophagy | 17 | 1.3E-2 |  |
| GO:0016241: Regulation of macroautophagy | 10 | 7.7E-2 |  |
| GO:0010506: Regulation of autophagy | 15 | 1.6E-1 |  |
| **Up-Regulated - Turquoise** | | | |
| **Annotation Cluster 5 - Enrichment Score: 1.7** | | | |
| **GOTERM_BP_FAT** | **Nº Genes** | **P value** | **Genes** |
| GO:0007033: Vacuole organization | 24 | 1.9E-3 | HAX1, HPS4, NBR1, NPC1, RAB24, RAB3GAP2, RAB3GAP1, RB1CC1, RRAGD, SUPT20H, UVRAG, VIPAS39, VPS37A, WDR45, XBP1, ATG16L2, CALCOCO2, CHMP1B, CHMP5, CLCN3, C9orf72, ERN1, EXOC4, FAM160A2, FNBP1L, GOLGA2, HOOK2, LRSAM1, KDM4A, MTMR3, PACS2, PIKFYVE, PLEKHF1, PRKAB2, PRKAG1, RNF41, SESN2, SCOC, SNX14, STX12, TMEM106B, TSG101, USP33 |
| GO:0016236: Macroautophagy | 23 | 4.8E-3 |  |
| GO:0006914: Autophagy | 35 | 2.1E-2 |  |
| GO:0016241: Regulation of macroautophagy | 12 | 1.1E-1 |  |
| GO:0010506: Regulation of autophagy | 20 | 1.4E-1 |  |
| **Down-Regulated - Grey60** | | | |
| **Annotation Cluster 2 - Enrichment Score: 11.79** | | | |
| **GOTERM_BP_FAT** | **Nº Genes** | **P value** | **Genes** |
| GO:1903047: Mitotic cell cycle process | 82 | 6.5E-13 | BUB1, BUB3, CNOT11, NAE1, NEK6, RAD1, RINT1, SKP1, TRIAP1, AURKAIP1, BIRC5, CALM2, CUL3, CCNB1, FEN1, HDAC8, NUSAP1, PIK3R4, PCNA, PSMC2, PSMC3, PSMD1, PSMD10, PSMD11, PSMD6, PSMD7, PSMD9, PSME3, PSMA2, PSMA3, PSMA4, PSMA5, PSMA6, PSMB1, PSMB2, PSMB5, RPA2, RPS27L, RNF4, TGFB1, UBE2C |
| GO:0022402: Cell cycle process | 101 | 6.7E-13 |  |
| GO:0000278: Mitotic cell cycle | 83 | 9.8E-12 |  |
| **Down-Regulated - Cyan** | | | |
| **Annotation Cluster 1 - Enrichment Score: 7.74** | | | |
| **GOTERM_BP_FAT** | **Nº Genes** | **P value** | **Genes** |
| GO:0000375: RNA splicing, via transesterification reactions | 45 | 9.6E-9 | ALYREF, CD2BP2, DDX1, DHX15, DDX39A, POLDIP3, ERCC3, FASTKD5, FUS, GRSF1, LEO1, MPHOSPH10, PHF5A, RTCB, RBM17, RBM4, POLR2F, POLR2H, SARNP, U2AF1, U2AF2, WDR77, ALKBH5, JMJD6, CACTIN, CSTF1, CSTF2, C1QBP, EIF4A3, HSPA1A, HSPA1B, HSPA8, HNRNPF, HNRNPH1, HNRNPM, MAGOHB, MAGOH, PPIL1, PDE12, PUF60, PRPF19, PRPF3, PRPF31, PRPF4, PDCD7, PPP1R8, SRSF1, SRSF4, SRSF9, SRRT, SNRPD1, SNRPD3, SNRNP40, SNRPC, SNRPE, SNRPG, SNRPB, SF3A3, SF3B4, USP39 |
| GO:0006397: mRNA processing | 58 | 1.3E-8 |  |
| GO:0000377: RNA splicing, via transesterification reactions with bulged adenosine | 44 | 2.0E-8 |  |
| GO:0000398: mRNA splicing, via spliceosome | 44 | 2.0E-8 |  |
| GO:0008380: RNA splicing | 52 | 3.7E-8 |  |

**Supplementary Table 4.** Somatic variants found in sequenced leukemic cell lines

| **Cell line** | **Chr** | **Position** | **Gene** | **Variant** | **VAF %** | **Mutation** | **COSMIC** |
| --- | --- | --- | --- | --- | --- | --- | --- |
| **THP-1** | **1** | 115256529 | NRAS | exon3:c.182A>T:p.Q61L | 7 | Nonsynonymous | COSM583 |
| **OCI-AML2** | **2** | 25466800 | DNMT3A | exon16:c.1903C>T:p.R635W | 9.8 | Nonsynonymous | COSM87012 |
|  | **17** | 7578235 | TP53 | exon2:c.218A>C:p.Y73S | 21.1 | Nonsynonymous | COSM215722 |
| **OCI-AML3** | **5** | 170837543 | NPM1 | exon10:c.772_773insTCTG:p.L258fs | 36.1 | Frameshift | COSM158604 |
|  | **1** | 115256529 | NRAS | exon3:c.182A>T:p.Q61L | 9.9 | Nonsynonymous | COSM583 |
|  | **2** | 25457243 | DNMT3A | exon23:c.2644C>T:p.R882C | 47.8 | Nonsynonymous | COSM1166704 |
| **HL-60** | **1** | 115256529 | NRAS | exon3:c.182A>T:p.Q61L | 69.6 | Nonsynonymous | COSM583 |
|  | **X** | 39934298 | BCOR | exon4:c.300dupA:p.G101fs | 66.5 | Frameshift | N/A |
